# Hidden folds reveal brain organization

**DOI:** 10.7554/eLife.111265

**Published:** 2026-04-15

**Authors:** Jürgen Germann

**Affiliations:** 1 Institute of Biomedical Engineering, University of Toronto Toronto Canada; 2 Krembil Brain Institute Toronto Canada; 3 Center for Advancing Neurotechnological Innovation to Application (CRANIA) Toronto Canada

**Keywords:** neuroanatomy, cortical folding, magnetic resonance imaging, occipital cortex, parietal cortex, spatial orientation, Human

## Abstract

Previously underappreciated folds in the cerebral cortex provide insight into how its structure varies across individuals.

**Related research article** Willbrand EH, Tsai YH, Gagnant T, Weiner KS. 2026. Updating the sulcal landscape of the human lateral parieto-occipital junction provides anatomical, functional, and cognitive insights. *eLife* **12**:RP90451. doi: 10.7554/eLife.90451.

As the brain grows during development, its outermost region – the cerebral cortex – folds to form a distinctive pattern of grooves and ridges (Chi et al., 1977). Although all human brains look slightly different, there are consistent features that have guided neuroscientists for more than a century. The largest folds, known as primary sulci, form during mid-gestation and are relatively consistent across individuals, while smaller folds develop later and are more variable (Demirci et al., 2023). This combination of consistency and variation is a defining feature of brain organization.

Many of the major folds seen in the human brain are conserved across primates, suggesting they reflect fundamental aspects of brain organization shaped over evolution (Amiez et al., 2019). For this reason, early studies used prominent sulci, such as the central sulcus, as landmarks for mapping function (Penfield and Rasmussen, 1950; Rasmussen and Milner, 1975). Smaller and more variable folds, however, have often been regarded as incidental rather than meaningful anatomical features (Ono et al., 1990).

Now, in eLife, Kevin Weiner (University of California, Berkeley) and colleagues – including Ethan Willbrand (University of Wisconsin-Madison) and Yi-Heng Tsai (University of North Carolina-Chapel Hill) as joint first authors – report that a region of the cerebral cortex once thought to have a simple folding pattern actually contains a number of previously overlooked sulci (Willbrand et al., 2026). This region, known as the lateral parieto-occipital junction, is involved in visual processing, attention and spatial thinking. By examining brain scans from a large group of individuals, the researchers identified four small sulci that are not included in standard anatomical descriptions of the brain. One of these folds stood out: the ventral supralateral occipital sulcus was present in almost every brain examined, suggesting it is a consistent anatomical feature rather than a rare variation. This highlights how even well-studied parts of the brain can contain underappreciated features.

Willbrand et al. then asked whether these folds carry meaningful information. These new sulci differed from neighboring folds in shape, thickness and degree of myelination, suggesting they mark distinct anatomical territories. Functional analyses further showed that the surrounding cortical areas are associated with specific brain networks. Variation in anatomical features was also related to behaviour: differences in sulcal depth were associated with performance on a spatial orientation task. This suggests that subtle aspects of cortical anatomy can indicate how functional systems are organized in individual brains.

These findings resonate with a long-standing view in neuroanatomy. Detailed anatomical studies suggest that cortical folding patterns reflect underlying organization (Amiez and Petrides, 2014; Petrides, 2011), with even small and variable sulci providing useful landmarks for interpreting brain function (Germann et al., 2020). Features such as branching patterns and interruptions, including buried gyral bridges known as *pli de passage* (Broca, 1888), can help identify corresponding regions across individuals even when their precise locations vary.

Advances in neuroimaging have progressively improved how the brain can be mapped (Figure 1). Surface-based approaches now incorporate cortical folding patterns, using measures such as sulcal depth and curvature to align brains more accurately across individuals (Van Essen et al., 1998). More recent work has refined these approaches by developing improved templates that better capture anatomical variability across individuals (Feilong et al., 2024). While these developments represent a major step forward, they do not fully capture the finest features of cortical anatomy. Work such as that by Willbrand and colleagues – who also include Thomas Gagnant (University of Bordeaux) – shows that additional structure remains to be resolved at smaller scales.

**Figure 1.**
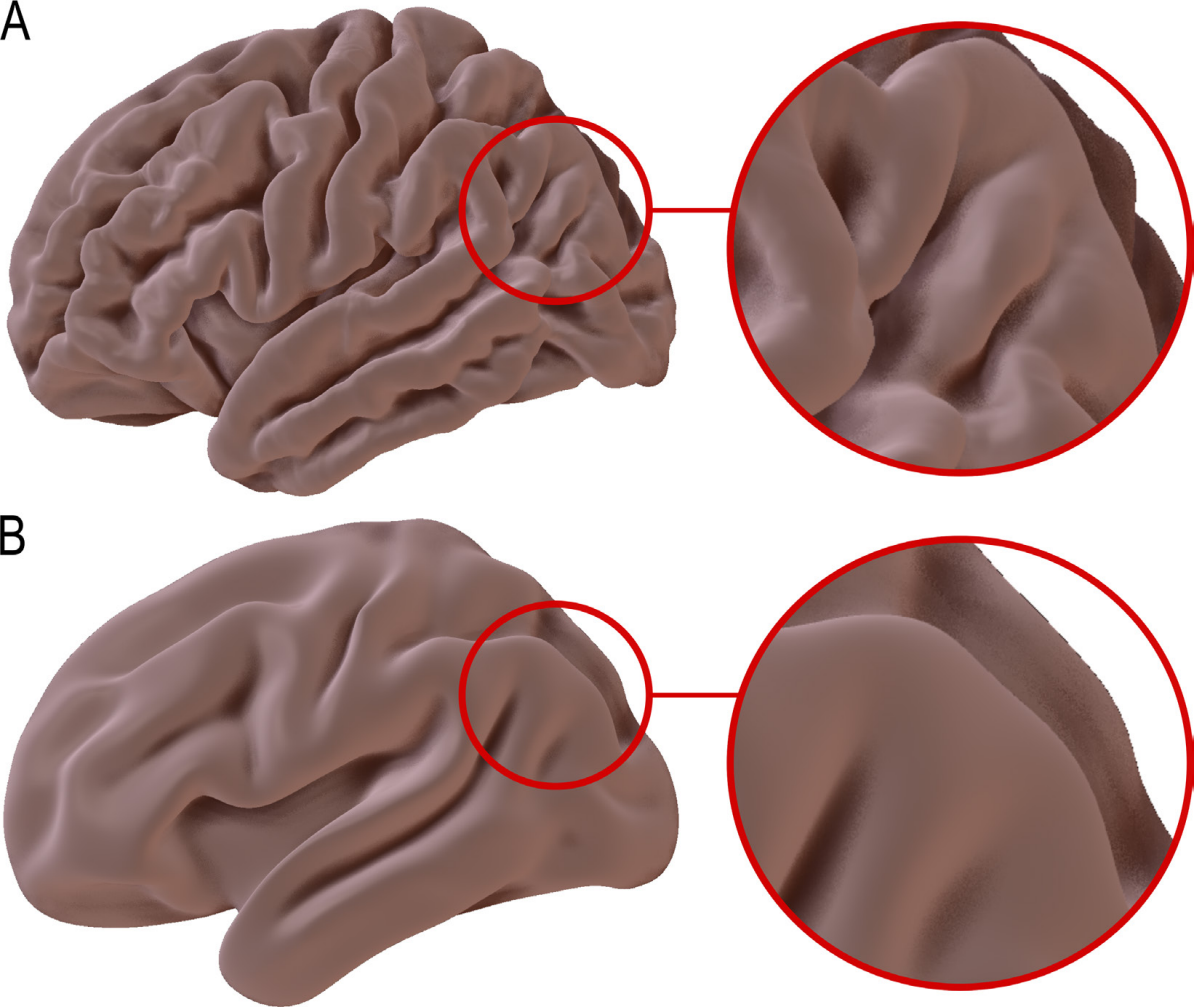
Individual variability and average representations of cortical folding. (**A**) A three-dimensional view of the left hemisphere from a single individual (the “Colin27” brain), reconstructed from magnetic resonance imaging (MRI). The cortical surface shows the complex and unique pattern of folds present in an individual brain. The inset illustrates fine-scale sulcal features within the lateral parieto-occipital junction. (**B**) A corresponding view of the left hemisphere of a population-averaged brain (MNI152), generated by aligning and averaging MRI data from many individuals using surface-based registration. This process preserves major sulci that are consistently located across individuals, while smaller and more variable folds are reduced or not retained. By examining brain scans from a large group of individuals, Willbrand et al. identified four small sulci within the lateral parieto-occipital junction that are not included in standard anatomical descriptions of the brain, including one that that was present in almost every brain examined, thus highlighting how well-studied parts of the cortex can contain previously unrecognized features. More information about Colin27 and MNI152 is available at https://mcin.ca/research/neuroimaging-methods/atlases/.

This raises an important question: how much of the brain’s organization remains hidden within these fine-scale features? Many cortical regions have not been examined with this level of anatomical precision, particularly the association cortex, where variability is high. It is therefore possible that additional sulci remain to be described, and that current maps of the brain are incomplete.

There are also practical challenges. Identifying small sulci requires detailed manual labelling, which limits the scale of such analyses. Automating the labelling process may allow these approaches to be applied to larger datasets, which would help researchers to determine how general these findings are, and to explore how anatomical variation relates to behaviour. Understanding brain organization is an ongoing process, with each methodological advance revealing structure at a finer scale.

From early anatomical descriptions to modern imaging, the field of neuroanatomy has moved toward increasingly precise representations of the cortex. Studies like the work of Willbrand et al. extend this trajectory by showing that even small, previously underappreciated folds can provide meaningful insight into brain organization. More broadly, this work highlights the importance of individual variability. Rather than being treated as noise, differences in cortical anatomy may reflect meaningful variation in how brain systems are arranged across people. Integrating fine-scale anatomical features with modern imaging approaches may therefore be an important step toward a more complete understanding of brain structure and function.
